# Supplementary material for: The inclusion of Amazon mangroves in Brazil’s REDD+ program
Source: Nat Commun. 2024 Mar 4;15:1549. doi: 10.1038/s41467-024-45459-w (PMC10912189; doi:10.1038/s41467-024-45459-w)

Supplementary Information for:

The inclusion of Amazon mangroves in Brazil's REDD+ program

Angelo F. Bernardino[1]*, Ana Carolina A. Mazzuco[1,9], Rodolfo F. Costa[2], Fernanda Souza[3], Margaret A. Owuor[4,5], Gabriel N. Nobrega[6], Christian J. Sanders[7], Tiago O. Ferreira[2] & J. Boone Kauffman[8]

[1]Departamento de Oceanografia, Universidade Federal do Espirito Santo, Av. Fernando Ferrari, 514, Goiabeiras, Vitória-ES. 29075-910, Brazil. [2]Department of Soil Science, Luiz de Queiroz College of Agriculture, University of Sao Paulo, Piracicaba, SP, Brazil. [3]Instituto Chico Mendes de Conservação da Biodiversidade, Ministério do Meio Ambiente, Macapá, AP, Brazil. [4]Wyss Academy for Nature at the University of Bern, Switzerland. [5]Institute of Ecology and Evolution, University of Bern, Switzerland. [6]Departamento de Ciências do Solo, Universidade Federal do Ceará, Fortaleza, CE, Brazil. [7]National Marine Science Centre, Southern Cross University, Coffs Harbour, NSW, 2540, Australia. [8]Department of Fisheires, Wildlife, and Conservation Sciences, Oregon State University, Corvallis, OR, United States. [9]Present address. UNESCO/IOC Project Office for IODE, Flanders Marine Institute, InnovOcean Campus, Oostende, Belgium. *email: angelo.bernardino@ufes.br

Supplementary Table 1. Transect metadata of mangrove plots in the studied sites on the Brazilian Amazon coast. N of plots – number of plots measured; Class.: Hydrogeomorphology – Estuarine, Delta, Delta/Open Coast. Na.: not available

Region	Site	N of plots	Date	Vegetation	Species dominance	Max soil depth (cm)	Soil pH	Soil Salinity	Soil Eh	Class	Lat (N)	Long (W)
Bragança	Barreto	6	Feb-17	Mangrove	*Rhizophora mangle, Avicennia germinans, Laguncularia racemosa*	300	6.2	17	na.	Estuarine	-0.745170	-48.011132
	Boca Grande	6	Feb-17	Mangrove		300	6.2	26	na.	Estuarine	-0.869444	-46.695556
	Caetano	6	Feb-17	Mangrove		300	6.4	17	na.	Estuarine	-0.749150	-48.013733
	Caete	6	Feb-17	Mangrove		300	6.2	20	na.	Estuarine	-0.981000	-46.722983
	Furo Chato	6	Feb-17	Mangrove		200	6.2	21	na.	Estuarine	-0.881217	-46.652300
	Furo Grande	6	Feb-17	Mangrove		300	6.5	18	na.	Estuarine	-0.841333	-46.638600
	Mangue Sul	6	Feb-17	Mangrove		300	6.5	21	na.	Estuarine	-0.918033	-46.679317
Maracanã	Maruipe	6	Feb-17	Mangrove		200	6.5	18	na.	Estuarine	-0.654933	-47.394233
	Salinas	6	Feb-17	Mangrove		270	6.5	18	na.	Estuarine	-0.632533	-47.367400
Curuça	Abade C	6	Jan-19	Mangrove		260	7	20.8	-245	Estuarine	-0.686194	-47.858667

	Abade D	6	Jan-19	Mangrove		190	7.3	13.5	-150	Estuarine	-0.685167	-47.853944
	Abade E	6	Jan-19	Mangrove		170	6	12.5	-160	Estuarine	-0.696139	-47.857500
	Ilha Ipomonga	6	Jan-19	Mangrove		300	5.8	12.2	12.7	Estuarine	-0.656500	-47.862778
	Muraja G	6	Jan-19	Mangrove		300	6.9	17.2	-78.8	Estuarine	-0.756778	-47.955389
	Ramos K	6	Jan-19	Mangrove		300	5.2	23.3	91.5	Estuarine	-0.736444	-47.998389
	Abade L	6	Jan-19	Mangrove		200	5.9	26.3	-69.2	Estuarine	-0.736437	-47.914759
	Bailique 1	6	Apr-22	Mangrove	Avicennia sp.	300	6.2	0.7		Delta/Open coast	0.870944	-50.014583
Bailique	Bailique 2	6	Apr-22	Mangrove	Pterocarpus sp., Rhizophora sp., Mauritia flexuosa, Avicennia sp.	300	6.0	0		Delta	0.907306	-50.032056
	Terra Grande 3	6	Apr-22	Mangrove	Pterocarpus sp., Mauritia flexuosa, Avicennia sp., Bamboos	300	4.6	0		Delta	1.063583	-49.957889
Araguari	Afuá Congo	6	Apr-22	Mangrove	Rhizophora sp., Avicennia sp., Aninga	100	4.6	0.5		Delta	1.153750	-49.922639

	Araguari 5	6	Apr-22	Mangrove	Rhizophora sp., Avicennia sp., Aninga	90	5.6	6.5		Delta/Open coast	1.166694	-49.891972
	Araguari 6	6	Apr-22	Mangrove	Pterocarpus sp., Avicennia sp., Rhizophora sp.	100	6.7	4.8		Delta/Open coast	1.155000	-49.894444
	Sucuriju 7	6	Apr-22	Mangrove	Rhizophora sp., Avicennia sp. (undersotry ferns)	300	5.6	9.8		Estuarine	1.681222	-49.934472
Sucuriju	Sucuriju 8	6	Apr-22	Mangrove	Rhizophora sp., Avicennia sp. (undersotry ferns)	300	5.1	1		Estuarine	1.684972	-49.962806
	Sucuriju 9	6	Apr-22	Mangrove	Rhizophora sp., Avicennia sp., Bamboo, (undersotry ferns)	300	5.0	0		Estuarine	1.670306	-49.964417
	Sucuriju 11	6	Apr-22	Mangrove	Rhizophora sp., Avicennia sp., Aninga	300	6.5	0		Delta/Open coast	1.690389	-49.925028
Bailique	Varzea Forest	6	Apr-22	Varzea forest	Aninga, Pterocarpus sp., Mauritia flexuosa, Bamboos	300	5.8	0		Delta	0.886242	-50.084667
Curuça	Pasture	6	Jan-19	Pasture		250	5.4	18.3	56.7	Estuarine	-0.737050	-47.915399
	Shrimp A	6	Jan-19	Shrimp pond		150	6.3	36.7	51.3	Estuarine	-0.686634	-47.857895

	Shrimp B	6	Jan-19	Shrimp pond	170	6.8	27.3	-65	Estuarine	-0.684460	-47.855493
	Shrimp H	6	Jan-19	Shrimp pond	180	5.9	13.8	94.2	Estuarine	-0.696100	-47.858358
	Shrimp I	6	Jan-19	Shrimp pond	150	5.9	30.7	60.1	Estuarine	-0.687262	-47.855812
Total		192									

Supplementary Table 2. Site level data on Total Ecosystem Carbon Stocks (TECS), Aboveground carbon (AGB) and Soil carbon (Soil C) of mangroves in the Brazilian Legal Amazon coast. Values given in Megagrams per hectare (Mg ha^{-1}) unless otherwise noted. Soil Organic Carbon (SOC) density given in depth-integrated top 1-m soils (mg.cm^{-3}). SE-Standard error.

Site	TECS	SOC dens. top 1m (mg.cm^{-3})	SE SOC	Total AGB	AGB SE	Total Soil C	Hydrogeomorphology
Barreto	501.1	17.2	1.5	104.8	18.9	382.1	Estuarine
Boca Grande	522.8	10.2	1.6	155.4	42.1	338.5	Estuarine
Caetano	746.2	19.4	2.6	223.4	64.5	490.1	Estuarine
Caete	520.7	16.7	1.2	121.8	37.3	371.3	Estuarine
Furo Chato	361.6	15.2	2.4	145.7	28.6	196.9	Estuarine
Furo Grande	457.8	12.8	1.0	96.5	19.2	331.8	Estuarine
Mangue Sul	543.0	14.9	1.1	194.8	48.5	310.3	Estuarine
Maruipe	408.9	18.0	2.4	147.6	17.0	246.7	Estuarine
Salinas	536.1	22.2	3.5	116.4	20.6	396.5	Estuarine
Abade C	524.9	23.7	2.7	65.9	26.7	435.2	Estuarine
Abade D	356.5	22.3	2.4	68.5	5.7	273.6	Estuarine
Abade E	387.7	16.5	3.0	54.9	11.1	309.4	Estuarine
Ilha Ipomonga	516.9	10.6	1.1	116.7	25.8	375.2	Estuarine
Muraja G	410.6	20.5	2.2	96.8	35.3	280.1	Estuarine
Ramos K	580.9	17.7	3.1	95.8	27.7	464.0	Estuarine
Abade L	435.6	13.8	2.8	127.0	28.4	288.3	Estuarine
Sucuriju 7	437.7	8.6	1.1	200.5	34.1	211.7	Estuarine
Sucuriju 8	538.6	12.1	1.9	230.8	48.7	287.3	Estuarine
Sucuriju 9	446.3	9.8	1.1	159.0	43.4	268.5	Estuarine
Bailique 2	902.6	22.9	4.1	335.0	47.2	508.1	Delta
Terra Grande 3	277.1	9.5	3.4	102.2	28.6	141.5	Delta
Afuá Congo	254.1	17.9	2.8	104.3	32.6	128.1	Delta
Araguari 5	215.7	11.7	1.8	86.5	30.6	89.5	Delta/open coast
Araguari 6	255.8	11.4	2.0	125.5	28.9	95.5	Delta/open coast
Bailique 1	180.9	4.4	0.7	29.4	4.7	103.8	Delta/open coast
Sucuriju 11	855.3	29.9	5.0	272.7	102.5	540.8	Delta/open coast
Varzea forest	604.4	14.6	2.7	288.5	80.8	153.1	Delta
Pasture	78.0	3.4	1.9			78.0	Pasture
Shrimp A	261.6	10.4	2.0			261.6	Shrimp pond
Shrimp B	96.4	4.6	1.2			96.4	Shrimp pond
Shrimp H	106.5	4.7	1.4			106.5	Shrimp pond
Shrimp I	70.7	4.6	1.6			70.7	Shrimp pond

Supplementary Table 3. PERMANOVA results comparing variability on in mangrove carbon stocks; Total soil carbon (C), Soil Organic Carbon (SOC) density top-1m, downed wood carbon (C), TAGC – Total Aboveground Carbon, TBGC – Total Belowground Carbon, TECS – Total Ecosystem Carbon Stocks, forest characteristics (tree basal area and density), and soil properties (soil salinity and bulk density) among hydrogeomorphic settings (estuarine, delta, delta/open coast). Note: soil salinity was log (x+10) transformed; p < 0.05 are indicated *.

**Total soil C**	**df**	**SS**	**MS**	**F**	***p***	**R^2**
Setting	2	0.51	0.25	6.93	0.02*	0.38
Residuals	23	0.84	0.04			0.62
Total	25	1.35				1.00

p < 0.05 pairwise results: estuarine > delta, estuarine > delta/open coast; delta > delta/open coast

**SOC density**	**df**	**SS**	**MS**	**F**	***p***	**R^2**
Setting	2	0.5	0.3	13.7	0.01*	0.11
Site	23	2.6	0.1	6.1	0.01*	0.57
Residuals	78	1.5	0.01			0.32
Total	103	4.6				1.00

p < 0.05 pairwise results for SOC density:
estuarine, delta > delta/open coast

**Downed wood C**	**df**	**SS**	**MS**	**F**	***p***	**R^2**
Setting	2	0.78	0.39	4.21	0.03*	0.27
Residuals	23	2.13	0.09			0.73
Total	25	2.91				1.00

p < 0.05 pairwise results: estuarine > delta

**TAGC**	**df**	**SS**	**MS**	**F**	***p***	**R^2**
Setting	2	0.08	0.04	0.72	0.49	0.06
Residuals	23	1.27	0.05			0.94
Total	25	1.35				1.00

**TBGC**	**df**	**SS**	**MS**	**F**	***p***	**R^2**
Setting	2	0.73	0.36	30.72	0.01*	0.73
Residuals	23	0.27	0.01			0.27
Total	25	1.00				1.00

p < 0.05 pairwise results: estuarine > delta, estuarine > delta/open coast

**TECS**	**df**	**SS**	**MS**	**F**	***p***	**R^2**
Setting	2	0.21	0.11	3.96	0.01*	0.26
Residuals	23	0.62	0.03			0.74
Total	25	0.83				1.00

p < 0.05 pairwise results: estuarine, delta > delta/open coast

**Tree basal area**	**df**	**SS**	**MS**	**F**	***p***	**R^2**
Setting	2	0.12	0.06	1.91	0.21	0.14
Residuals	23	0.73	0.03			0.86
Total	25	0.85				1.00

**Tree density**	**df**	**SS**	**MS**	**F**	***p***	**R^2**
Setting	2	1.80	0.90	12.13	0.01*	0.51
Residuals	23	1.71	0.07			0.49
Total	25	3.51				1.00

p < 0.05 pairwise results: estuarine ≠ delta, estuarine ≠ delta/open coast

**Soil salinity**	**df**	**SS**	**MS**	**F**	***p***	**R^2**
Setting	2	0.10	0.05	16.1	0.01*	0.58
Residuals	23	0.07	0.003			0.42
Total	25	0.18				1.00

p < 0.05 pairwise results: estuarine > delta, estuarine > delta/open coast

**Bulk density**	**df**	**SS**	**MS**	**F**	***p***	**R^2**
Setting	2	0.01	0.01	0.60	0.59	0.05
Residuals	23	0.25	0.01			0.95

Total	25	0.27		1.00

Supplementary Table 4. Linear regression for the relationship of total ecosystem carbon stocks (TECS) in mangroves of the Amazon coast based on variables possible to collect via remote sensing (tree basal area), weather stations (precipitation), or simple field measurements (salinity and latitude). ** significant at p=0.01

**Coefficients**	**Estimate**	**Std. Error**	**T value**	**Pr(>|t|)**
Intercept	238.36034	498.93691	0.478	0.63777
Precipitation	-0.07945	0.24962	-0.318	0.75342
Soil salinity	8.50940	4.36665	1.949	0.06481
Latitude	5.85814	122.15499	0.048	0.96220
Tree basal area	9.76060	2.70232	3.612	0.00164**
**Residual error**	**Degrees of freedom**	**F-statistic**	**Adjusted R^2**	
146.1	21	3.77	0.3071	

Supplementary Table 5. Best multiple regression model based on Akaike Information Criterion (AIC) for the relationship of total ecosystem carbon stocks in mangroves of the Amazon coast with tree basal area and soil salinity. * significant at p=0.05; *** significant at p=0.001

**Coefficients**	**Estimate**	**Std. Error**	**T value**	**Pr(>|t|)**
Intercept	79.218	103.067	0.769	0.4499
Soil salinity	8.447	3.496	2.416	0.0240 *
Tree basal area	9.435	2.363	3.993	0.00057***
**Residual error**	**Degrees of freedom**	**F-statistic**	**Adjusted R^2**	
139.9	21	8.158	0.364	

Supplementary Table 6. PERMANOVA results for soil isotopic $\delta^{13}C$ signatures between ecosystems (mangroves, varzea forests and pasture/shrimp ponds), and hydrogeomorphology settings (estuarine, delta, and delta open coast). Data was log (x+100) transformed. Tests with a p value < 0.05 are highlighted *.

**δ13C**	**df**	**SS**	**MS**	**F**	***p***	**R^2**
Ecosystem	3	0.001	0.0005	58.21	0.01*	0.22
Residuals	627	0.005	0.00001			0.78
Total	640	0.006				1.00

p < 0.05 pairwise results: Mangrove ≠ shrimp pond ≠ pasture ≠ varzea forest

**δ13C**	**df**	**SS**	**MS**	**F**	***p***	**R^2**
Settings	4	0.002	0.005	60.12	0.01*	0.28
Residuals	626	0.005	0.000007			0.72
Total	630	0.006				1.00

p < 0.05 pairwise results: shrimp ≠ estuarine ≠ pasture ≠ delta, delta open coast

Supplementary Table 7. PERMANOVA results comparing land use change (shrimp ponds and pasture) with paired pristine mangrove forests. Total soil carbon (C), Soil Organic Carbon (SOC) density top-1m, downed wood carbon (C), TAGC – Total Aboveground Carbon, TBGC – Total Belowground Carbon, TECS – Total Ecosystem Carbon Stocks, and soil bulk density. Downed wood C and TAGC were log (x+10) transformed. Tests with a p value < 0.05 are highlighted *.

**Total soil C**	**df**	**SS**	**MS**	**F**	***p***	**R^2**
Land-use	1	0.48	0.48	13.09	0.03*	0.65
Residuals	7	0.26	0.04			0.36
Total	8	0.73				1.00
**SOC density**	**df**	**SS**	**MS**	**F**	***p***	**R^2**
Land-use	1	0.7	0.7	39.3	0.02*	0.85
Residuals	7	0. 1	0.02			0.15
Total	8	0.8				1.00
**Downed wood C**	**df**	**SS**	**MS**	**F**	***p***	**R^2**

Land-use	1	0.03	0.03	38.08	0.02*	0.84
Residuals	7	0.01	0.0009			0.16
Total	8	0.04				1.00
**TAGC**	**df**	**SS**	**MS**	**F**	***p***	**R^2**
Land-use	1	0.22	0.22	406.43	0.02*	0.98
Residuals	7	0.004	0.0005			0.02
Total	8	0.23				1.00
**TBGC**	**df**	**SS**	**MS**	**F**	***p***	**R^2**
Land-use	1	0.04	0.04	464.31	0.01*	0.99
Residuals	7	0.0006	0.0001			0.01
Total	8	0.04				1.00
**TECS**	**df**	**SS**	**MS**	**F**	***p***	**R^2**
Land-use	1	0.68	0.68	19.59	0.01*	0.74
Residuals	7	0.24	0.03			0.26
Total	8	0.93				1.00
**Bulk density**	**df**	**SS**	**MS**	**F**	***p***	**R^2**
Land-use	1	0.29	0.29	19.29	0.02*	0.73
Residuals	7	0.10	0.01			0.24
Total	8	0.39				1.00

Supplementary Table 8. Forest Reference Level (FREL) for Mangroves in the Brazilian Amazon between years of 2016 and 2021, indicating average deforestation rates (activity - LULCC), and potential emission reductions (Mg CO_2e) based on the mean EF (1227.5 Mg CO_2e ha^{-1}).

	Legal Amazon Mangrove Deforestation		REDD+ credits (Mg CO_2e)	
	Mean (ha yr^{-1})	95% CI	Annual mean	Propagated error
Amazon Mangrove Reference Level (2016-2021)	751.3	248.7		
Best case scenario 100% mitigation			922,221	10,813

Supplementary Figures

Supplementary Figure 1. Transect-level total Ecosystem Carbon Stocks of Amazon mangroves. TAGC – Total Aboveground Carbon, TBG – Total Belowground carbon, Downed wood – Total downed wood carbon, Total soils – Total soil carbon. (a) Average carbon stocks of mangroves in the Legal Brazilian Amazon; (b) Average carbon stocks of mangroves on the coast of Amapá state, Northern Brazil; (c) Average carbon stocks of mangroves on the coast of Pará state, Northern Brazil. All values given in Megagrams (Mg) of carbon per hectare (ha). Error bars are 1 standard deviation

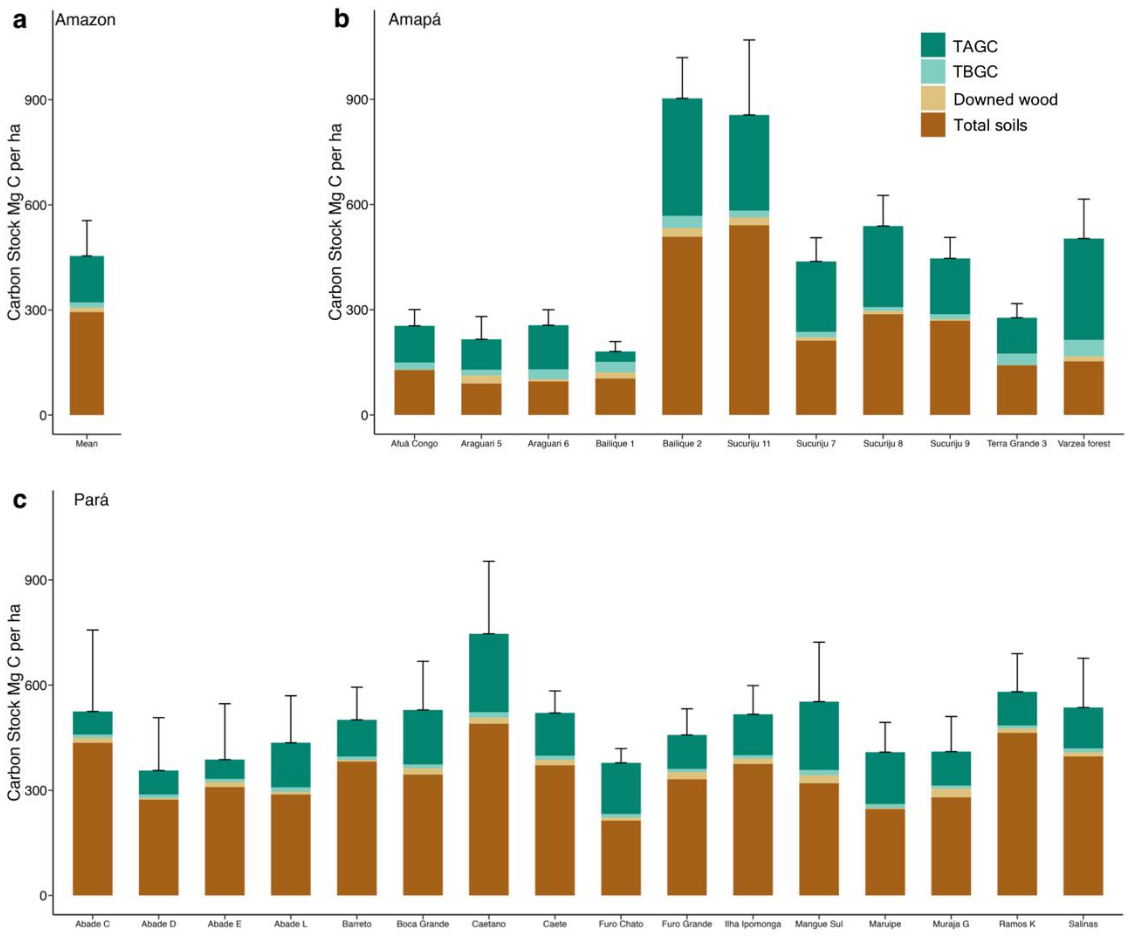

Supplementary Figure 2. Regression of Amazon mangrove TECS – Total Ecosystem Carbon Stocks with (a) soil salinity and (b) tree basal area (square meter per hectare); based on the best fitted model (Adj R^2=0.364; Equation: Y= 79.22 + 8.45 * soil salinity + 9.4 * basal area). Lower ribbon bars represent the data points used in the analysis. Shaded area represents the 95% CI; all values given in Megagrams (Mg) of carbon per hectare (ha).

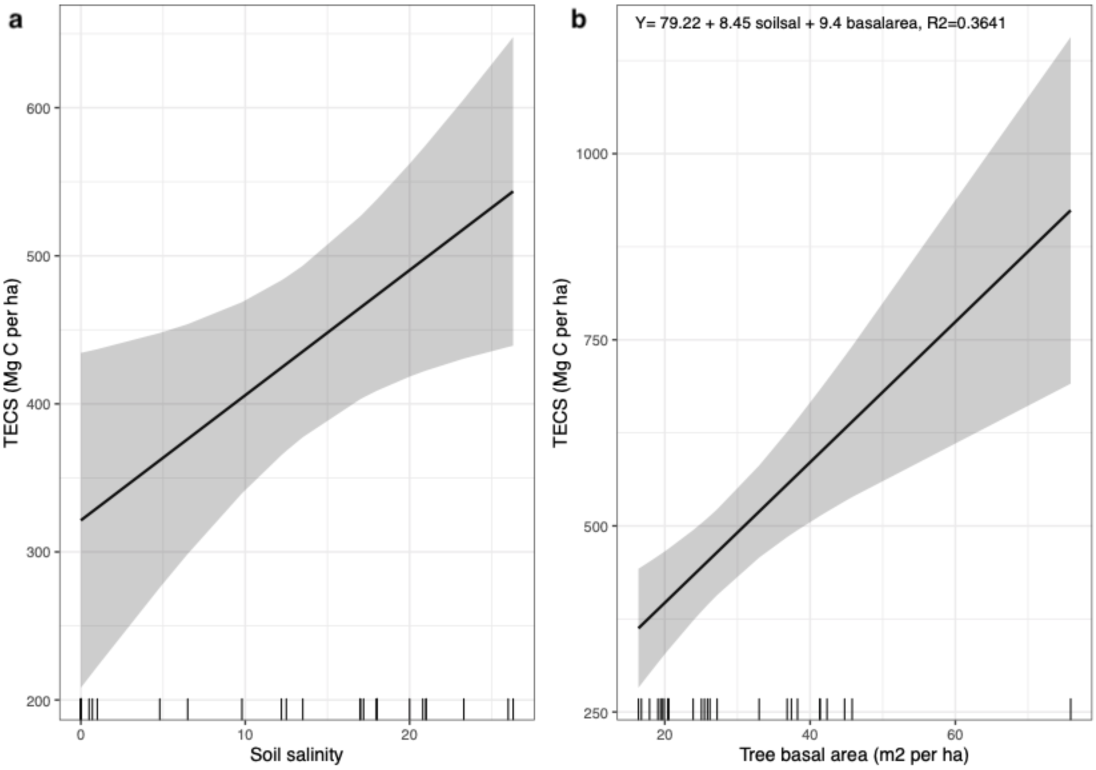

Supplementary Figure 3. Stable isotope signatures (deltaC^{13}) and C/N ratio of soil samples from the Amazon mangroves (N=914). Standard Ellipse Areas plotted according to hydrogeomorphic classification of mangroves and impacted sites (shrimp farms and pasture). Range signatures (Delta C^{13} x C/N ratio) of main organic sources, including Marine Particulate Organic Carbon (Marine POC), Marine Dissolved Organic Carbon (Marine DOC), Freshwater Particulate Organic Carbon (Freshwater POC), Freshwater Dissolved Organic Carbon (Freshwater DOC), and Mangrove plant material (Mangrove plants) indicated in colored boxes.

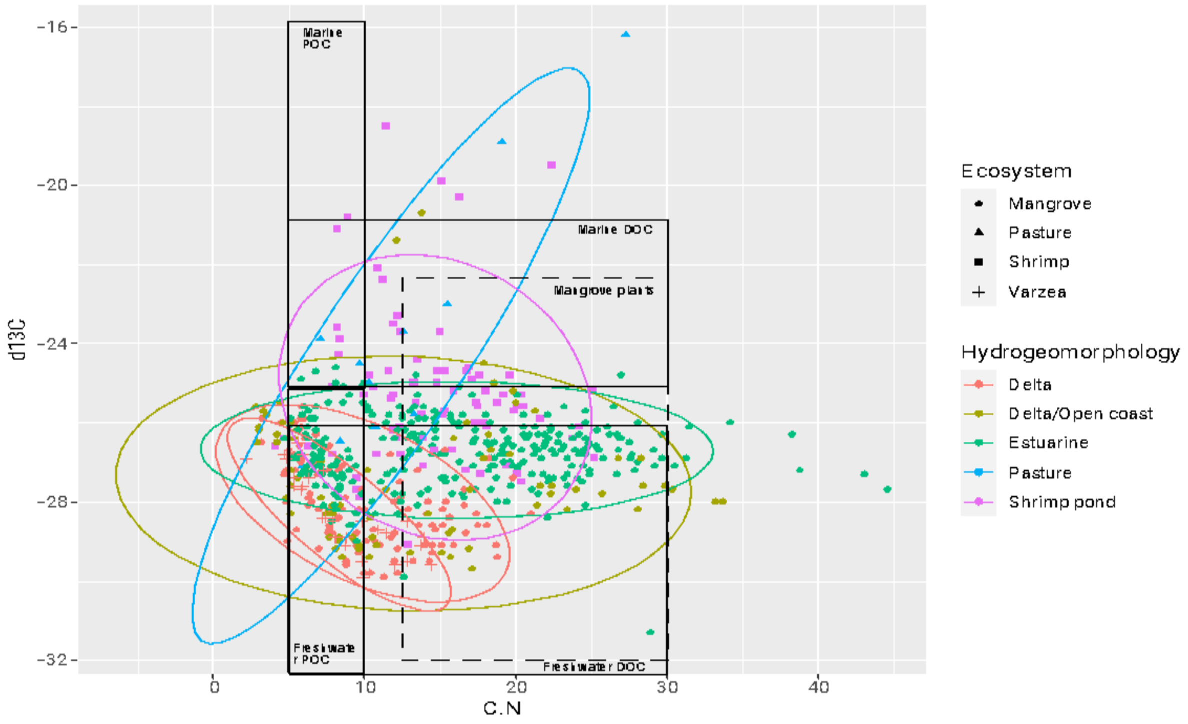

Supplementary Figure 4. Soil properties in mangrove areas converted to shrimp ponds and pastures in the Legal Amazon. (a) Soil bulk density, (b) Carbon (C) content, and (c), Soil Organic Carbon (SOC) density. Boxplots bars represent the lower quartile, median and upper quartile, wiskers indicate minimum and maximum values, and dots indicate data points above or below 1.5x the quartiles.

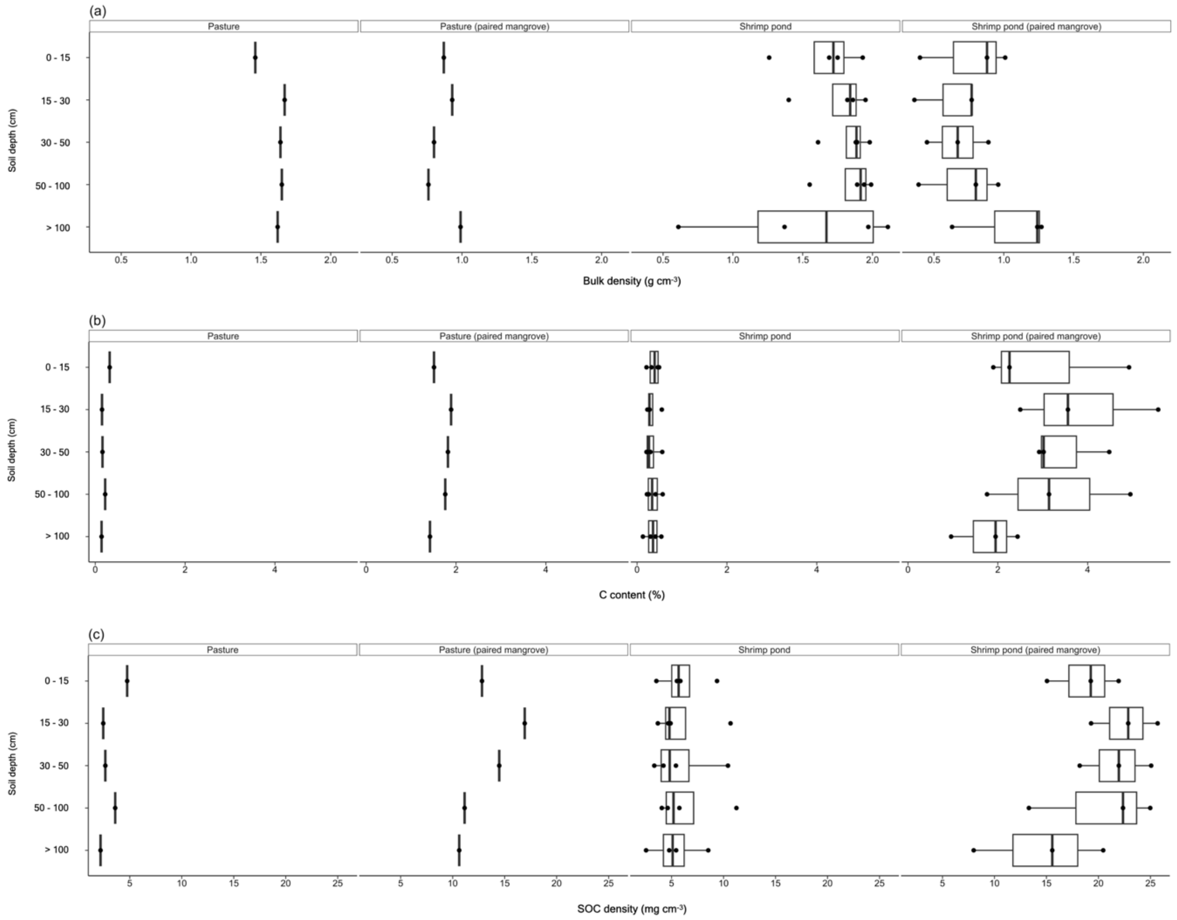

Supplement: Supplementary file 1 — Supplementary Information [file 41467_2024_45459_MOESM1_ESM.pdf]
